# Evaluating Risk of Neglect Among Older Adults Using NSHAP Round 3 (2015-2016)

**DOI:** 10.1093/geroni/igab046.1058

**Published:** 2021-12-17

**Authors:** Melissa Howe, Grey Pierce, Selena Zhong, Lissette Piedra, Won Choi

**Affiliations:** 1 NORC at the University of Chicago, Chicago, Illinois, United States; 2 University of Chicago, Chicago, Illinois, United States; 3 University of Illinois at Urbana-Champaign, Urbana, Illinois, United States

## Abstract

As functional health declines, dependency on others increases along with the risk of neglect and its harmful consequences. In this paper, we use data collected during 2015-16 (Round 3) of the National Social Life, Health, and Aging Project (NSHAP) to identify older adults at risk of neglect and to test the hypothesis that high neglect risk predicts poorer health. Specifically, we use NSHAP’s functional health survey module and follow-up care receiving “loop” to categorize respondents as having either “high” or “low” neglect risk. NSHAP’s functional health module assesses respondents’ difficulties with Activities of Daily Living (ADLs) and Instrumental Activities of Daily Living (IADLs). Because ADLs and IADLs are integral to the maintenance of physical health, hygiene, and well-being, the unrequited desire for help with such activities could signal neglect. Accordingly, we assign “high neglect risk” to respondents who report either that they: (a) want but are not getting help with an ADL or IADL; or (b) are getting help with an ADL or IADL, but from a helper who is not very reliable. Motivated by current research that documents higher rates of morbidity and mortality among neglected older adults, we examine associations between neglect risk and other key NSHAP measures, including indicators of physical health, mental health, cognition, social support, social strain, and field interviewer assessed respondent hygiene. Results suggest that this method of risk assessment can be useful in identifying vulnerable populations of older adults. Follow-up interviews are needed to further confirm its utility as a risk assessment tool.

